# Supplementary material for: Predictive analyses of regulatory sequences with EUGENe
Source: Nat Comput Sci. 2023 Nov 16;3(11):946–56. doi: 10.1038/s43588-023-00544-w (PMC10768637; doi:10.1038/s43588-023-00544-w)
Supplement: Supplementary file 1 — Supplementary Figs. 1–5 and Table 1. [file 43588_2023_544_MOESM1_ESM.pdf]

nature computational science

Brief Communication

https://doi.org/10.1038/s43588-023-00544-w

Predictive analyses of regulatory sequences with EUGENe

In the format provided by the authors and unedited

nature computational science

Brief Communication

https://doi.org/10.1038/s43588-023-00544-w

Predictive analyses of regulatory sequences with EUGENe

In the format provided by the authors and unedited

Supplementary Information

Supplementary Figure 1. STARR-seq plant promoter activity prediction. **2**
Supplementary Figure 2. RNA binding protein (RBP) specificity prediction. **4**
Supplementary Figure 3. JunD binding classifier interpretation. **5**
Supplementary Figure 4. Peak memory usage and batch processing time for datasets with increasing numbers of 10,000bp sequences. **7**
Supplementary Figure 5. Implementing custom architectures and training tasks in EUGENe. **8**
Supplementary Table 1. Common deep learning for regulatory genomics tasks can be run end-to-end with EUGENe **9**

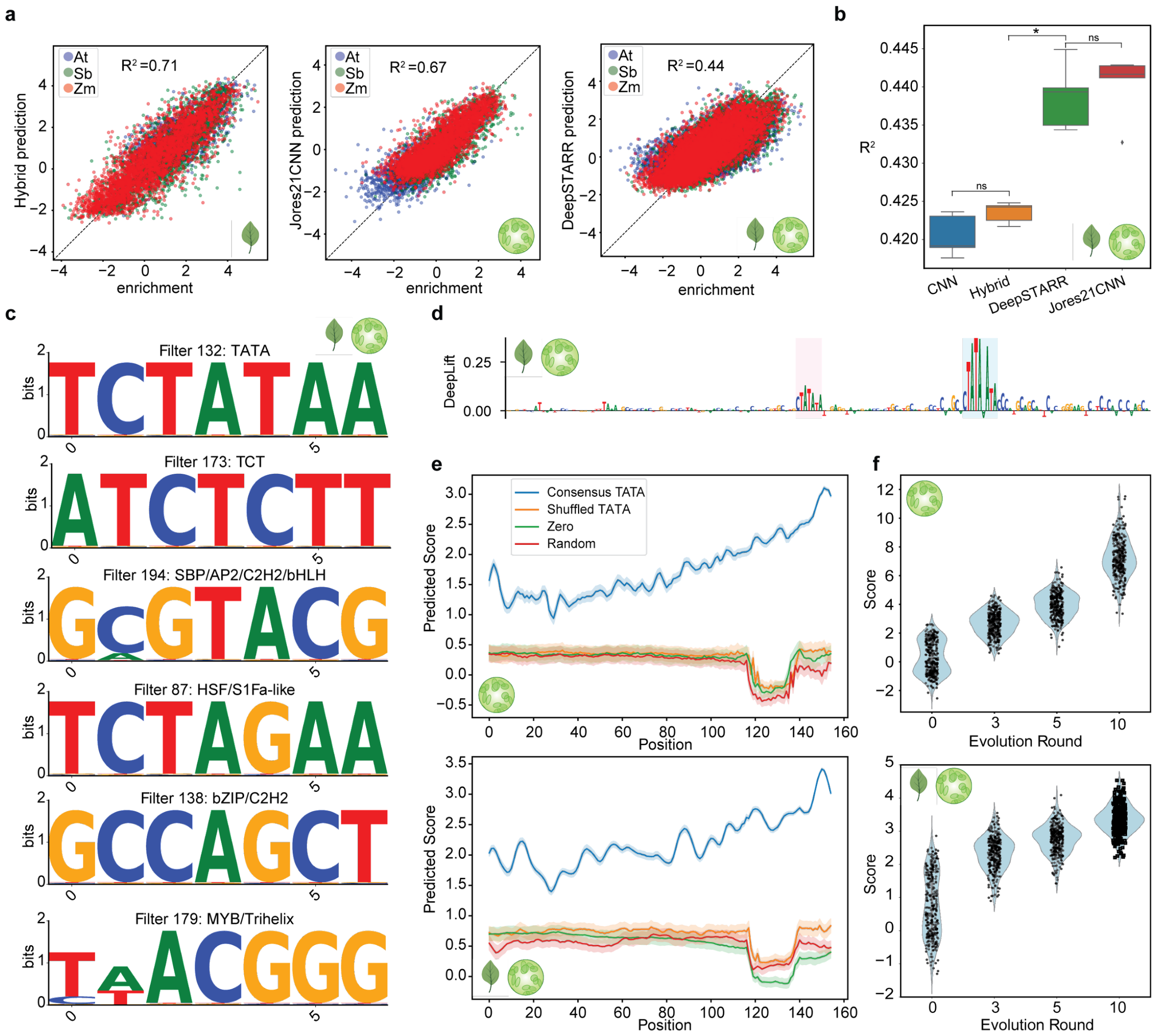

Supplementary Figure 1. STARR-seq plant promoter activity prediction.

a, Performance scatterplots colored by species of origin for the best leaf (left), protoplast (middle) and combined (right) models. **b,** Predictive performance of all trained combined models. The boxplots show distributions of R^2 values on held-out test data for each architecture across n=5 independent experiments (random initializations). The boxes show medians along with low and high quartiles. Whiskers extend to the furthest datapoint within 1.5 times the interquartile range. More extreme points are marked as outliers. A two-sided Mann-Whitney U test was used to determine p-values which were adjusted by the Benjamini-Hochberg method (* = $p < 0.05$, ns = not significant). Test statistics and adjusted p-values were: CNN-Hybrid (u=4, adjusted p-value=0.11), CNN-DeepSTARR (u=0, adjusted p-value=0.01), CNN-Jores21CNN (u=0, adjusted p-value=0.01), Hybrid-DeepSTARR (u=0, adjusted p-value=0.01), Hybrid-Jores21CNN (u=0, adjusted p-value=0.01), DeepSTARR-Jores21CNN (u=16, adjusted p-value=0.55). **c,** PWMs for a hand-selected set of learned combined model filters (not initialized with known PWMs). **d,** Attribution scores calculated using the DeepLIFT method for the sequence with the highest predicted value in the best DeepSTARR combined model **e,** Best

Jores21CNN protoplast (top) and DeepSTARR combined (bottom) model scores for n=310 sequences with an implanted consensus TATA box motif, shuffled consensus TATA box motif, all zeros motif, and random motif at every possible position. Mean model scores with 95% confidence intervals are shown. **f,** Model scores for the same set of n=310 promoters at different rounds of evolution compared against baseline (0) for the best protoplast (top) and combined (bottom) model.

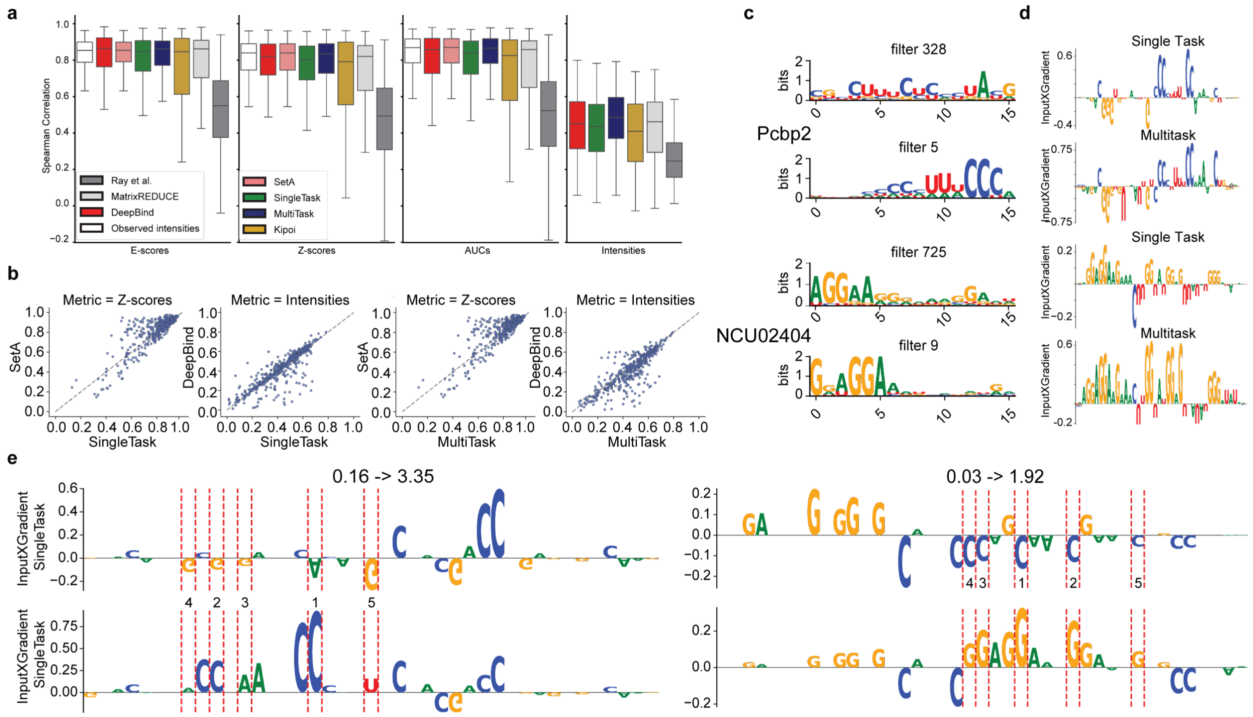

Supplementary Figure 2. RNA binding protein (RBP) specificity prediction.
a, Spearman correlations across four different metrics with each metric calculated from comparisons between observed (Set B) and predicted binding intensities (see **Methods** for more details on how each metric is calculated). Each boxplot indicates a distribution of Pearson correlations across all n=244 RBPs, except for Kipoi which includes n=89 RBPs. Ray et al, MatrixREDUCE, DeepBind and Observed intensities refer to correlations calculated from predicted intensities reported in Alipanahi *et al*. Observed intensities and SetA refer to correlations calculated using the intensities from Set A probes as the predicted intensities (see **Methods**). The boxes show medians along with low and high quartiles. Whiskers extend to the furthest datapoint within 1.5 times the interquartile range. **b,** Performance comparison scatterplots for the indicated models and metrics. Each dot indicates a comparison of the Pearson correlation between two models on a single RBP. **c,** Multitask and single task filters with TomTom significant annotations for Pcbp2 (top) and NCU02404 (bottom). **d,** The feature attributions calculated using the InputXGradient method for single task and multitask models using the sequence with the highest observed intensity in the test set for Pcbp2 (top) and NCU02404 (bottom). **e,** Two more examples of InputXGradient attribution scores for random (top row) and evolved (bottom row) sequences after evolution with the Pcbp2 (left) and NCU02404 (right) single task models. Red dashed lines indicate mutations made during evolution annotated with the round the mutation occurred in.

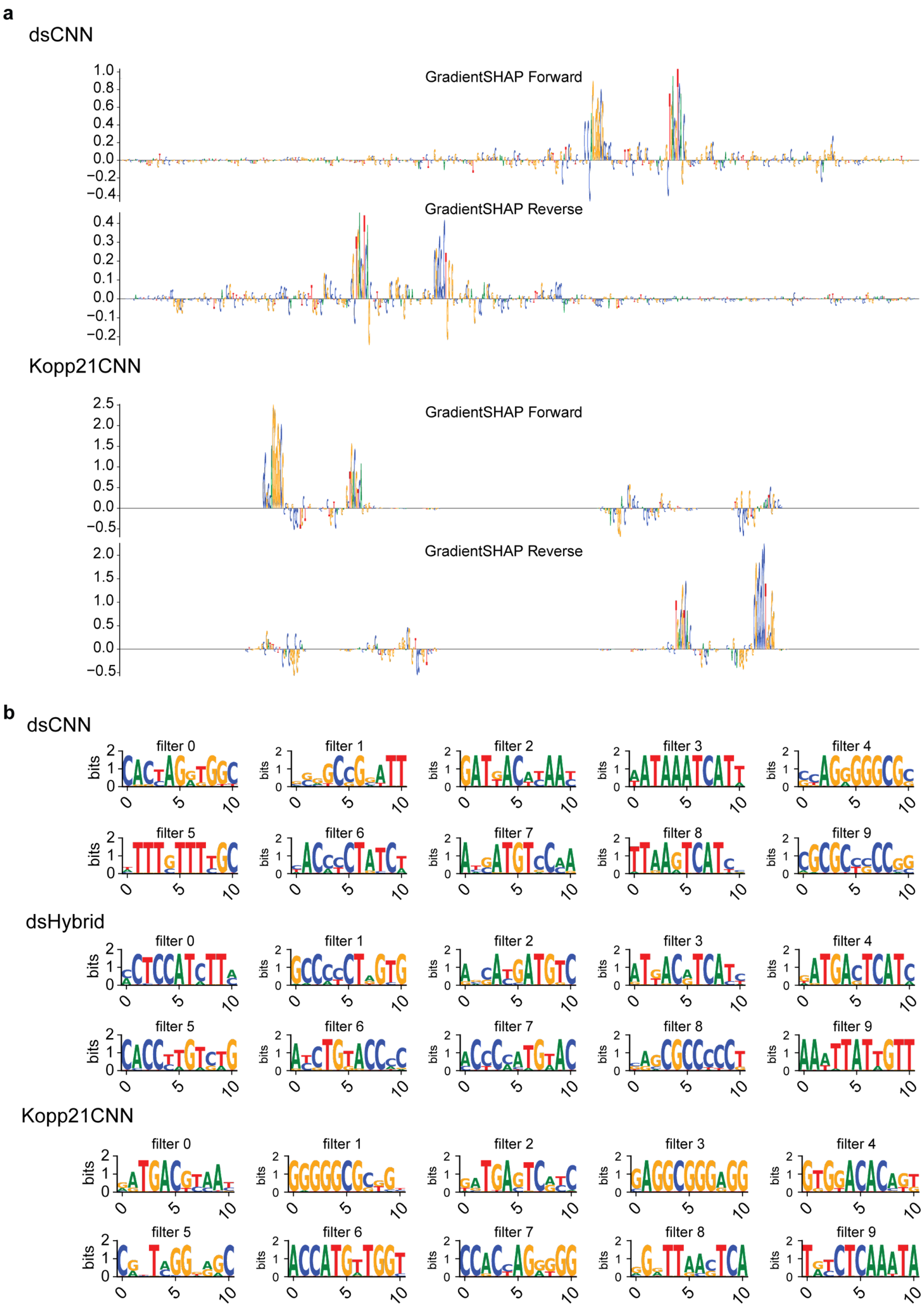

Supplementary Figure 3. JunD binding classifier interpretation.

a, Attribution scores calculated using GradientSHAP for the forward and reverse complement of the sequence with the highest predictions in each of the dsCNN and Kopp21CNN models. **b,** PWM visualizations of the 10 filters for the three convolutional architectures trained for JunD

binding classification.

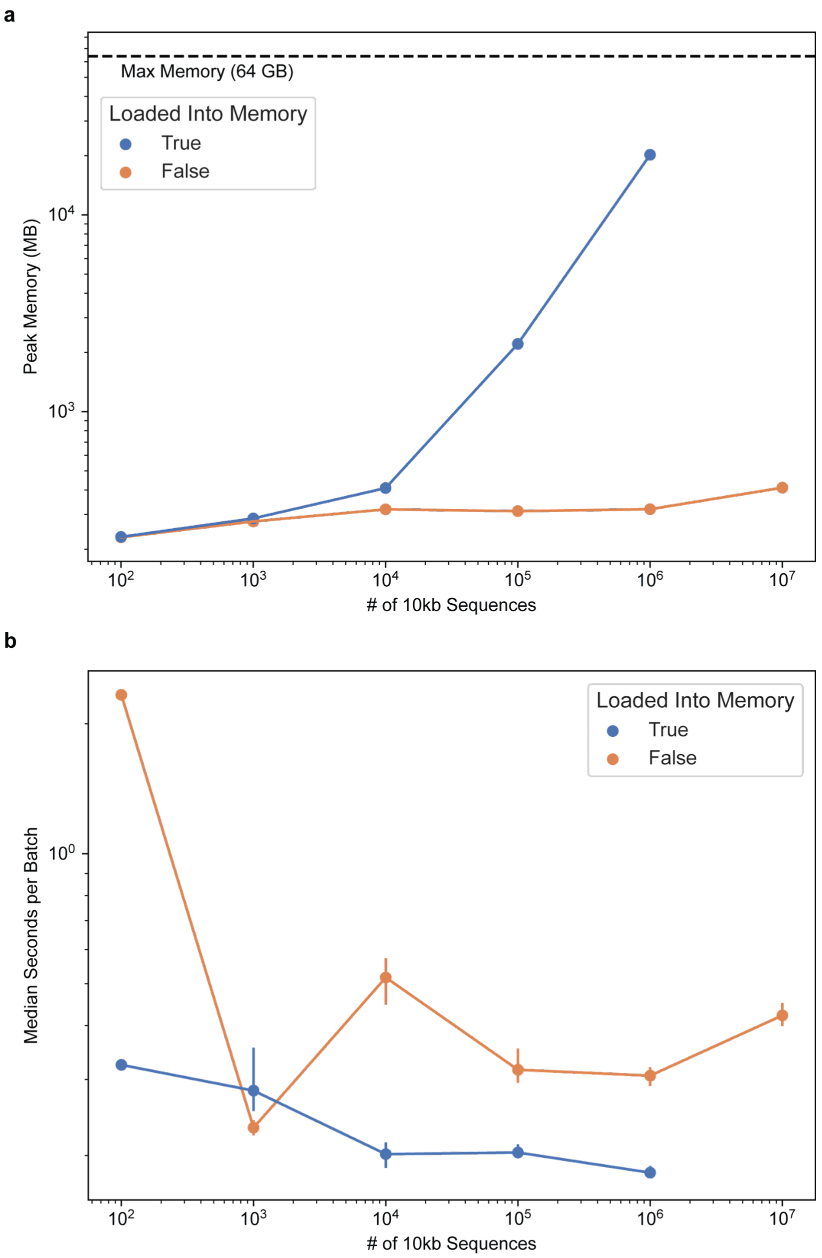

Supplementary Figure 4. Peak memory usage and batch processing time for datasets with increasing numbers of 10,000bp sequences.

a, Peak memory usage against number of sequences for datasets loaded into memory versus datasets loaded out-of-core. **b,** Median time in seconds taken for processing a batch of 100 sequences using the same datasets as in **a.** Error bars indicate interquartile ranges across up to 100 random batches. Both axes in **a** and **b** are on the $\log_{10}$ scale. All analyses were performed using a chunk size of 4096 along the sequence dimension on a machine with 2 CPU cores and 64GB of RAM.

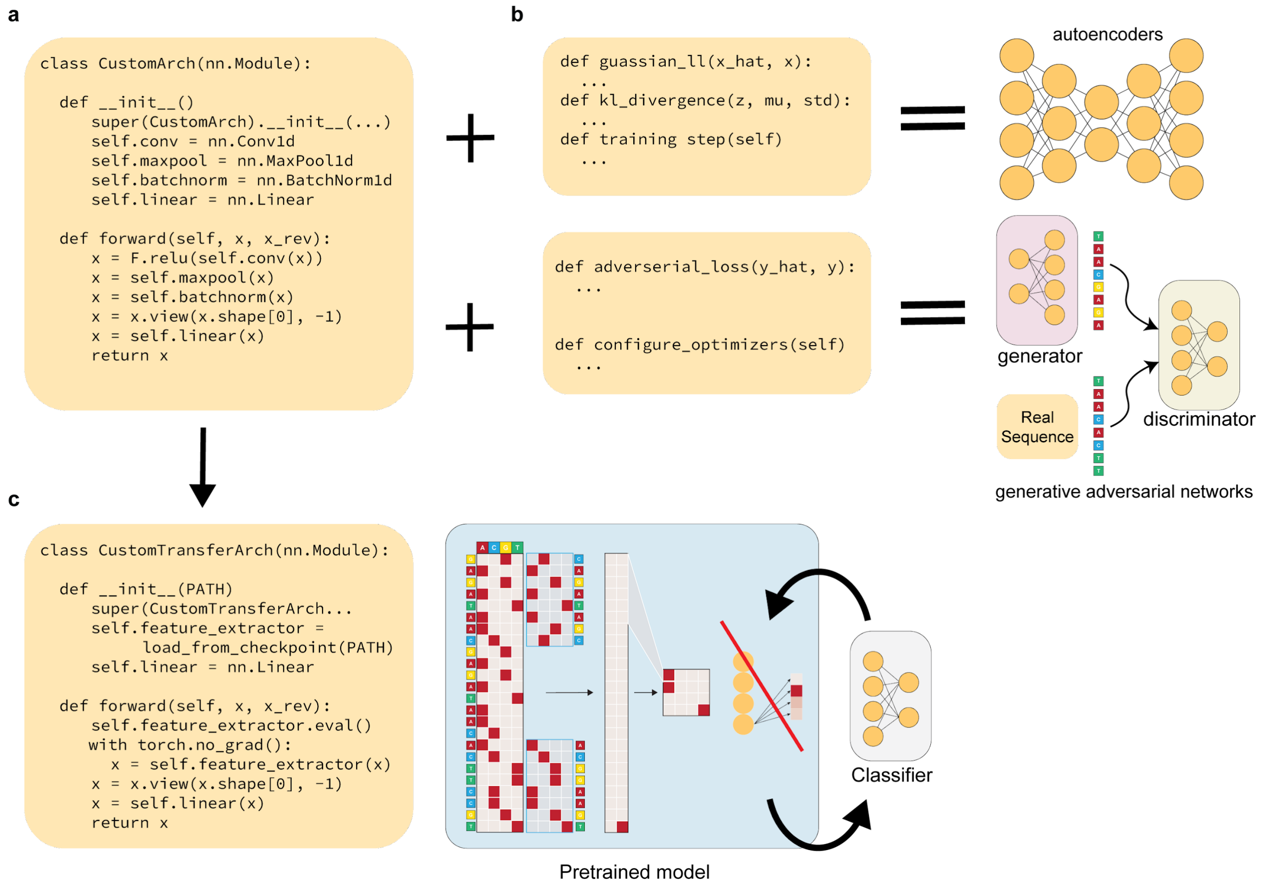

Supplementary Figure 5. Implementing custom architectures and training tasks in EUGENe.

a, Creating custom architectures that are compatible with EUGENe's training protocol involves first inheriting from the torch.nn.Module class, then defining the model's layer composition (__init__) and the forward propagation (forward) method. **b**, Architectures can also be wrapped in LightningModules to allow for training protocols other than EUGENe's current built-ins. For instance, a variational autoencoder, or VAE, requires creating two functions for calculating different parts of the loss and implementing how the functions are integrated into the training function (in its most basic form). We have omitted the changes needed to define an encoder and decoder structure and how that is handled in forward. A generative adversarial network (GAN), as another example, requires implementing a multipart loss function and configuring multiple optimizers to handle the training of the generator and discriminator. **c,** Transfer learning from pretrained models is also possible in EUGENe, and can be accomplished with simple changes to an architectures initialization and forward functions. Namely, a pretrained PyTorch model needs to be loaded in the __init__ method and then utilized in the forward method.

Supplementary Table 1. Common deep learning for regulatory genomics tasks can be run end-to-end with EUGENe

Task	Examples	Potential insights gained	ETL	Training and evaluation	End-to-end currently available?	Interpretation analyses currently available	Example in EUGENe use cases
Single task regression from a tabular file	DeepBind, ResidualBind	Identification and quantification of motif importance on continuous or binary events (e.g. RBP binding)	Yes	Yes	Yes	Filter interpretation, attribution analysis, evolution, GIA	DeepBind
Single track classification of peak regions from a single bed file	DeepBind	Identification and quantification of motif importance on binary events (e.g. TF binding)	Yes	Yes	Yes	Filter interpretation, attribution analysis, evolution, GIA	DeepBind
Multitask track classification (ChIP, ATAC, DNase, etc.) of peak regions from multiple bed files	DeepSEA, DanQ, Basset, Sei, Satori[26]	Identification and quantification of motif importance on biochemical activity (e.g. TF binding, transcription, DNA accessibility, etc.) Variant effects on biochemical activity	Yes	Yes	Yes	Filter interpretation, attribution analysis, evolution, GIA	Basset
Multitask track regression (ChIP, ATAC, DNase, etc.) at binned or base-pair resolution	Basenji, Enformer, BPNet	Identification and quantification of motif importance on biochemical activity (e.g. transcription, DNA accessibility, etc. Variant effects on biochemical activity CRE syntax rules	Yes	Yes	Yes	Filter interpretation, GIA	BPNet
Single task and multitask CRE activity prediction (both regression and classification (multiclass and multilabel)	DeepSTARR, MPRA-DragoNN	Identification and quantification of motif importance on CRE activity Variant effects on CRE activity CRE syntax rules	Yes	Yes	Yes	Filter interpretation, attribution analysis, evolution, GIA	DeepSTARR
Single cell ATAC-seq topic classification (multiclass classification)	DeepMEL, DeepMEL2, DeepFlyBrain	Identification and quantification of cell type specific motif importance Cell type specific variant effect prediction Cell type specific CRE syntax	Requires preproces-sing with pycisTopic	Yes	Yes, with preprocessing performed by pycisTopic	Filter interpretation, attribution analysis, evolution, GIA	DeepMEL
Single cell ATAC-seq cell accessibility prediction*	scBasset	Single cell analysis (e.g. denoising) Identification and quantification of cell type specific motif importance	Requires preproces-sing with ScanPy	Yes	Yes, with preprocessing performed by ScanPy	Filter interpretation, attribution analysis, evolution, GIA	scBasset
