# Supplementary material for: Predictive analyses of regulatory sequences with EUGENe
Source: Nat Comput Sci. 2023 Nov 16;3(11):946–56. doi: 10.1038/s43588-023-00544-w (PMC10768637; doi:10.1038/s43588-023-00544-w)
Supplement: Supplementary file 2 — Reporting Summary [file 43588_2023_544_MOESM2_ESM.pdf]

nature portfolio

Corresponding author(s): Hannah Carter

Last updated by author(s): Sep 19, 2023

Reporting Summary

Nature Portfolio wishes to improve the reproducibility of the work that we publish. This form provides structure for consistency and transparency in reporting. For further information on Nature Portfolio policies, see our Editorial Policies and the Editorial Policy Checklist.

Statistics

For all statistical analyses, confirm that the following items are present in the figure legend, table legend, main text, or Methods section.

n/a	Confirmed	
☐	☒	The exact sample size (*n*) for each experimental group/condition, given as a discrete number and unit of measurement
☐	☒	A statement on whether measurements were taken from distinct samples or whether the same sample was measured repeatedly
☐	☒	The statistical test(s) used AND whether they are one- or two-sided<br>*Only common tests should be described solely by name; describe more complex techniques in the Methods section.*
☐	☒	A description of all covariates tested
☐	☒	A description of any assumptions or corrections, such as tests of normality and adjustment for multiple comparisons
☐	☒	A full description of the statistical parameters including central tendency (e.g. means) or other basic estimates (e.g. regression coefficient) AND variation (e.g. standard deviation) or associated estimates of uncertainty (e.g. confidence intervals)
☐	☒	For null hypothesis testing, the test statistic (e.g. *F*, *t*, *r*) with confidence intervals, effect sizes, degrees of freedom and *P* value noted<br>*Give P values as exact values whenever suitable.*
☒	☐	For Bayesian analysis, information on the choice of priors and Markov chain Monte Carlo settings
☒	☐	For hierarchical and complex designs, identification of the appropriate level for tests and full reporting of outcomes
☐	☒	Estimates of effect sizes (e.g. Cohen's *d*, Pearson's *r*), indicating how they were calculated

Our web collection on statistics for biologists contains articles on many of the points above.

Software and code

Policy information about availability of computer code

Data collection	All datasets used in this study are publicly available and were collected from the sources listed in their respective publications.
Data analysis	The EUGENe source code is available on GitHub at https://github.com/ML4GLand/EUGENe and as a package on PyPi at https://pypi.org/project/eugene-tools/. Code that utilizes the package to produce the results presented in the manuscript can be found on GitHub at https://github.com/ML4GLand/EUGENe_paper. For the results presented and discussed in the manuscript, we used EUGENe v0.1.2. Other software versions for packages mentioned in the manuscript include:<br><br>Captum v0.5.0<br>Dask v2023.3.24<br>MotifData v0.0.1<br>PyTorch Lightning v2.0.0<br>SeqData v0.0.1<br>SeqDatasets v0.0.1<br>SeqExplainer v0.0.1<br>SeqPro v0.1.3<br>PyTorch v2.0.0<br>Xarray v2023.4.0<br>Yuzu v4.1.1<br>Zarr v2.14.2<br>modisco-lite v2.0.7<br>bpnet-lite v0.5.1

bedtools v2.31.0
Kipoi v0.8.6
ushuffle v1.1.2
RayTune 2.4.0
Seaborn v0.12.2
Matplotlib v3.6.2
Numba v0.57.0
NumPy v1.23.5
Pandas v1.5.2
TomTom v5.5.4
LogoMaker v0.0.8

For manuscripts utilizing custom algorithms or software that are central to the research but not yet described in published literature, software must be made available to editors and reviewers. We strongly encourage code deposition in a community repository (e.g. GitHub). See the Nature Portfolio guidelines for submitting code & software for further information.

Data

Policy information about availability of data

All manuscripts must include a data availability statement. This statement should provide the following information, where applicable:

- Accession codes, unique identifiers, or web links for publicly available datasets
- A description of any restrictions on data availability
- For clinical datasets or third party data, please ensure that the statement adheres to our policy

All datasets used in this study are publicly available. Raw and processed data for the plant promoter STARR-seq were obtained from https://github.com/tobjores/Synthetic-Promoter-Designs-Enabled-by-a-Comprehensive-Analysis-of-Plant-Core-Promoters. Normalized RNA probe binding intensities were obtained from http://hugheslab.ccbr.utoronto.ca/supplementary-data/RNAcompete_eukarya/norm_data.txt.gz. JunD peaks from human embryonic stem cells (H1-hesc) called with the hg38 reference genome were obtained from encodeproject.org (ENCFF446WOD, conservative IDR thresholded peaks, narrowPeak format). Blacklisted regions for hg38 were obtained from http://mitra.stanford.edu/kundaje/akundaje/release/blacklists/hg38-human/hg38.blacklist.bed.gz. TomTom queries were performed against the Ray2013 Homo sapiens and the HOCOMOCO v11 FULL motif collections for the RBP binding and JunD binding use cases respectively. The JunD PFM was obtained from https://jaspar.genereg.net/matrix/MA0491.1 for the in silico implantation experiment. 89 RBP models were obtained from the Kipoi mode repository at https://kipoi.org/models/DeepBind/Homo_sapiens/RBP/. We have also deposited the EUGENe specific dataset files and trained models used in the analyses presented here on Zenodo (Klie 2023). These represent the processed data files and SeqData objects that can be used along with the accompanying code to generate the figures for all the use cases. Source data for Figure 2, Extended Data Figure 1, and Extended Data Figure 2 is available with this manuscript.

Human research participants

Policy information about studies involving human research participants and Sex and Gender in Research.

Reporting on sex and gender	not applicable
Population characteristics	not applicable
Recruitment	not applicable
Ethics oversight	not applicable

Note that full information on the approval of the study protocol must also be provided in the manuscript.

Field-specific reporting

Please select the one below that is the best fit for your research. If you are not sure, read the appropriate sections before making your selection.

☒ Life sciences ☐ Behavioural & social sciences ☐ Ecological, evolutionary & environmental sciences

For a reference copy of the document with all sections, see nature.com/documents/nr-reporting-summary-flat.pdf

Life sciences study design

All studies must disclose on these points even when the disclosure is negative.

Sample size	The three datasets used in the manuscript represent diverse data types and modeling tasks that highlight core aspects of EUGENe and showcase its flexibility. We replicated analyses with the same sample sizes as previously reported, which were sufficient to train complex machine learning models for analysis of these datasets
Data exclusions	No data were excluded from these analyses.

Replication	All models were trained across 5 random initializations to assess reproducibility. We observed successful replication across the 5 trials in all three datasets.
Randomization	Training, validation, and test sets for training models were generated via random splits of the full datasets or as described in previous publications when applicable.
Blinding	Investigators were not blinded to group allocation during data analysis as knowledge of group allocation was necessary for training models.

Reporting for specific materials, systems and methods

We require information from authors about some types of materials, experimental systems and methods used in many studies. Here, indicate whether each material, system or method listed is relevant to your study. If you are not sure if a list item applies to your research, read the appropriate section before selecting a response.

Materials & experimental systems

n/a	Involved in the study	
☒	☐	Antibodies
☒	☐	Eukaryotic cell lines
☒	☐	Palaeontology and archaeology
☒	☐	Animals and other organisms
☒	☐	Clinical data
☒	☐	Dual use research of concern

Methods

n/a	Involved in the study	
☒	☐	ChIP-seq
☒	☐	Flow cytometry
☒	☐	MRI-based neuroimaging
